# Supplementary material for: A novel chimeric coronavirus spike vaccine combining SARS-CoV-2 RBD and scaffold domains from HKU-1 elicits potent neutralising antibody responses
Source: NPJ Vaccines. 2025 Nov 28;10:275. doi: 10.1038/s41541-025-01323-6 (PMC12753774; doi:10.1038/s41541-025-01323-6)
Supplement: Supplementary file 1 — Supplementary Information [file 41541_2025_1323_MOESM1_ESM.pdf]

Supplementary Figures

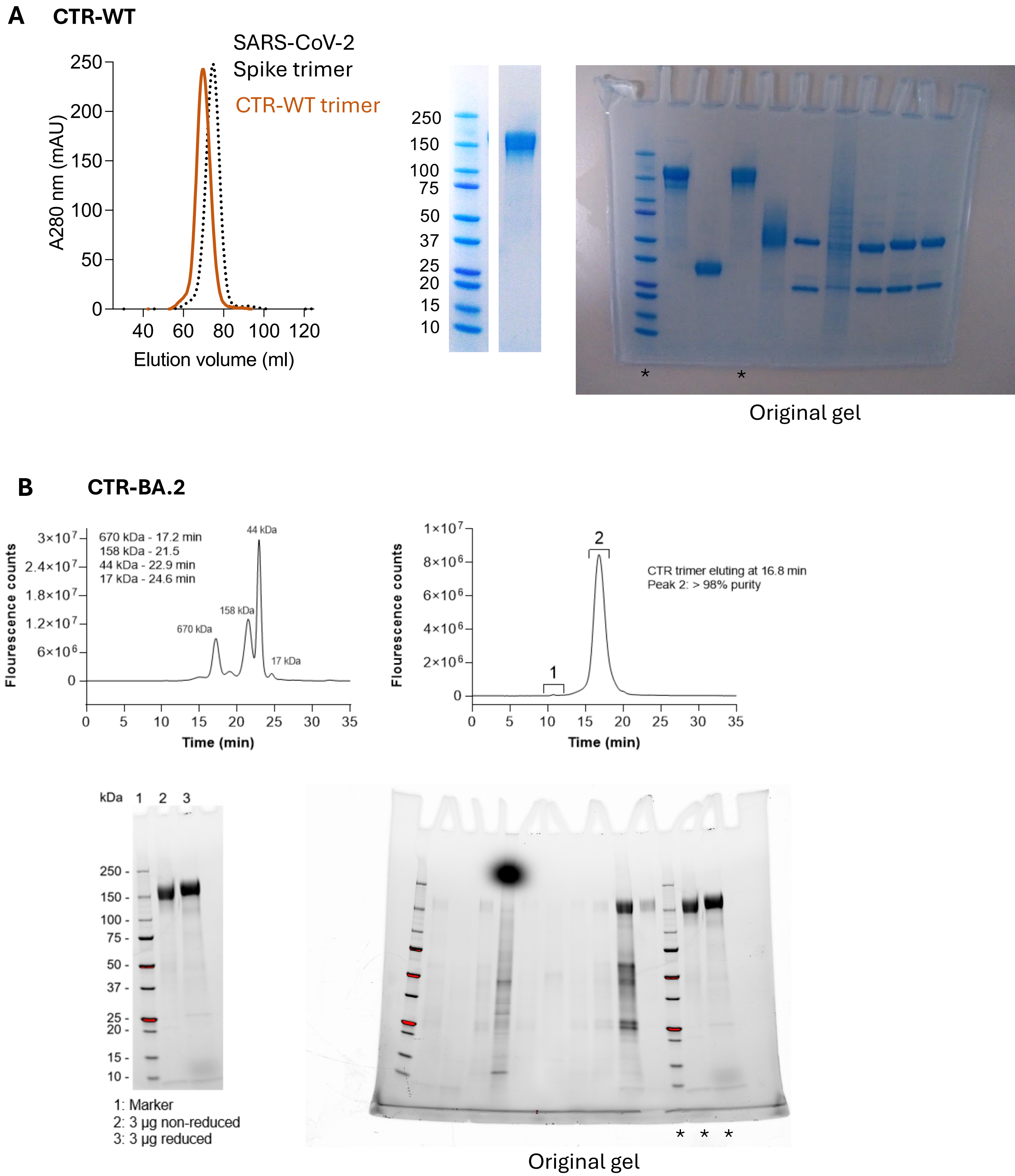

Figure S1. Validation of purified chimeric trimer RBD glycoproteins. Affinity/size exclusion chromatography trace and expected size shown by SDS-PAGE for **(A)** CTR-WT and **(B)** CTR-BA.2 glycoproteins. * demarcates lanes of original SDS-PAGE gels cropped for clarity.

A

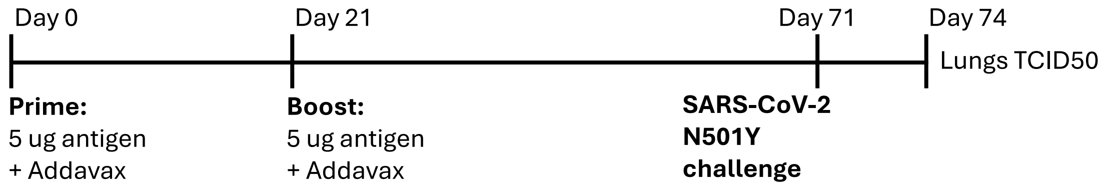

Prime day 0	Boost day 21
OVA	OVA
Spike Wu-1 (Ancestral/WT)	Spike Wu-1 (Ancestral/WT)
Spike Wu-1 (Ancestral/WT)	CTR-WT
CTR-WT	CTR-WT
Spike HKU-1	CTR-WT

B

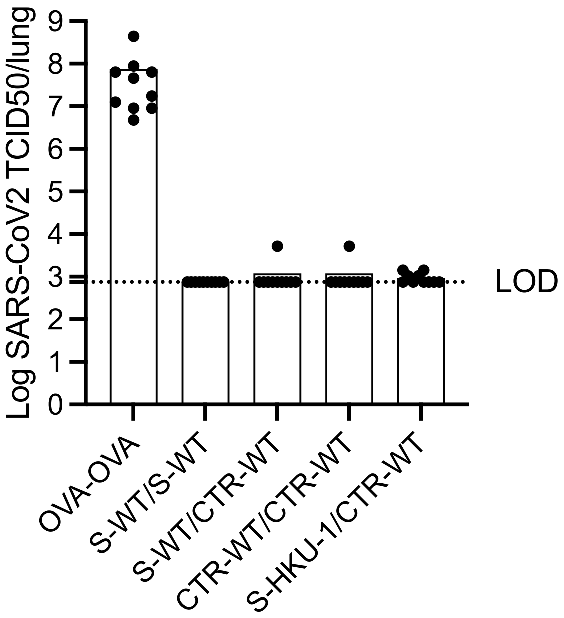

Figure S2. Immune challenge of BALB/c mice with mouse infectious SARS-CoV-2 virus. (A) Immunisation schedule and challenge with SARS-CoV-2 N501Y virus. **(B)** Viral load measurement of SARS-CoV-2 N501Y virus in lungs of mice three days post-challenge. Dotted lines denote limit of detection.

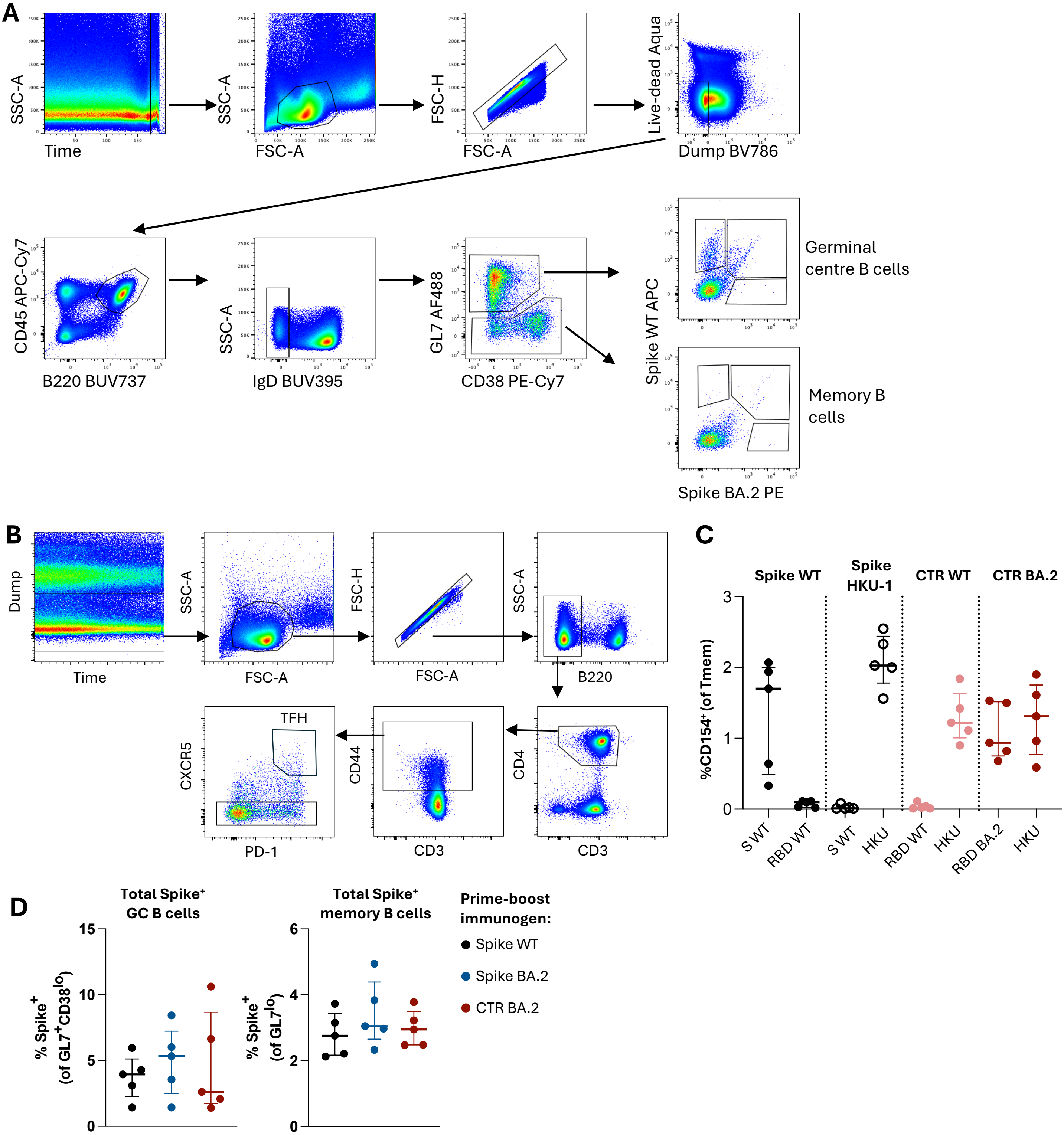

Figure S3. Identification of B cells and CD4 T cells in mice. (A) B cells were identified by SSC-A vs time, FSC-A vs SSC-A, followed by doublet exclusion (FSC-A vs FSC-H). Live and $CD3^-$ $F4/80^-$ $streptavidin^-$ (dump channel) cells were gated and $CD45^+$ $B220^+$ IgD^- B cells identified. Germinal centre ($GL7^+$ $CD38^{lo}$) or memory ($GL7^{lo}$) B cells were then assessed for binding to SARS-CoV-2 spike WT and/or BA.2 probes. **(B)** Gating strategy for lymph node CD4 TFH or bulk Tmem cells, based on $CD44^{hi}$, PD-1 and CXCR5 expression. **(C)** Frequencies of antigen-specific $CD154^+$ bulk Tmem cells upon restimulation with whole spike or RBD proteins. **(D)** Frequencies of total spike-specific GC and memory B cells at day 105 in mice after vaccination schedule in Figure 4A.

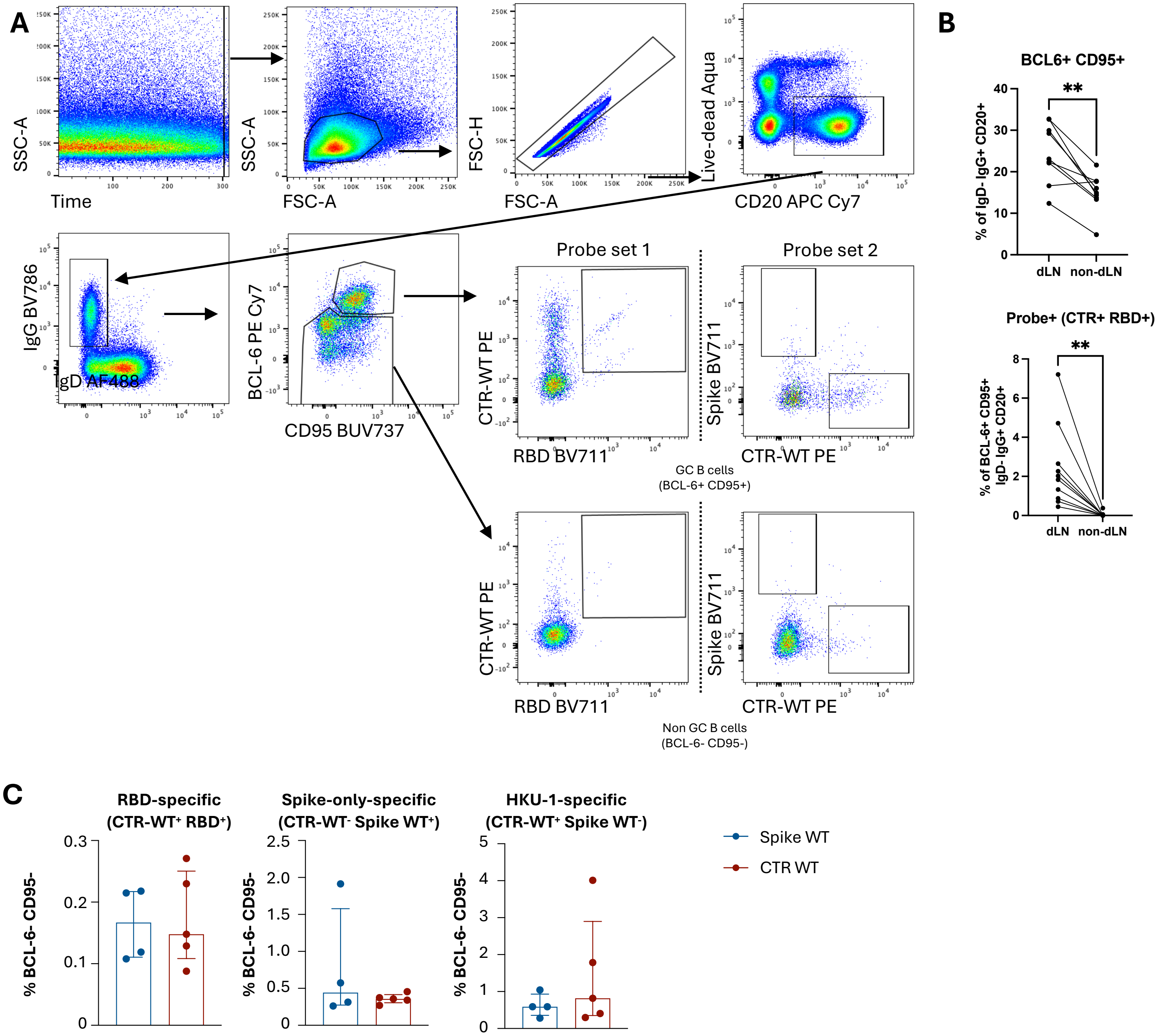

Figure S4. Identification of lymph node B cells in macaques. (A) B cells were first identified by SSC-A vs Time, FSC-A vs SSC-A gating, followed by doublet exclusion (FSC-A vs FSC-H). Cells were then gated on dump$^-$ (CD3$^-$ CD8$^-$ CD14$^-$ CD10$^-$ CD16$^-$ streptavidin$^-$) live CD20$^+$ B cells. GC and non-GC B cells were identified from class-switched IgD$^-$ B cells with BCL-6$^+$ CD95^{++} or BCL-6^{lo} staining, respectively. Antigen-specific cells were identified using two probe combinations: CTR-WT/RBD WT or spike WT/CTR-WT. **(B)** Comparison of total and antigen-specific GC B cell frequencies between paired draining and non-draining lymph nodes of vaccinated macaques. ** P < 0.01, statistics assessed by Wilcoxon test. **(C)** Frequency of antigen-specific non-GC B cells in draining lymph nodes.

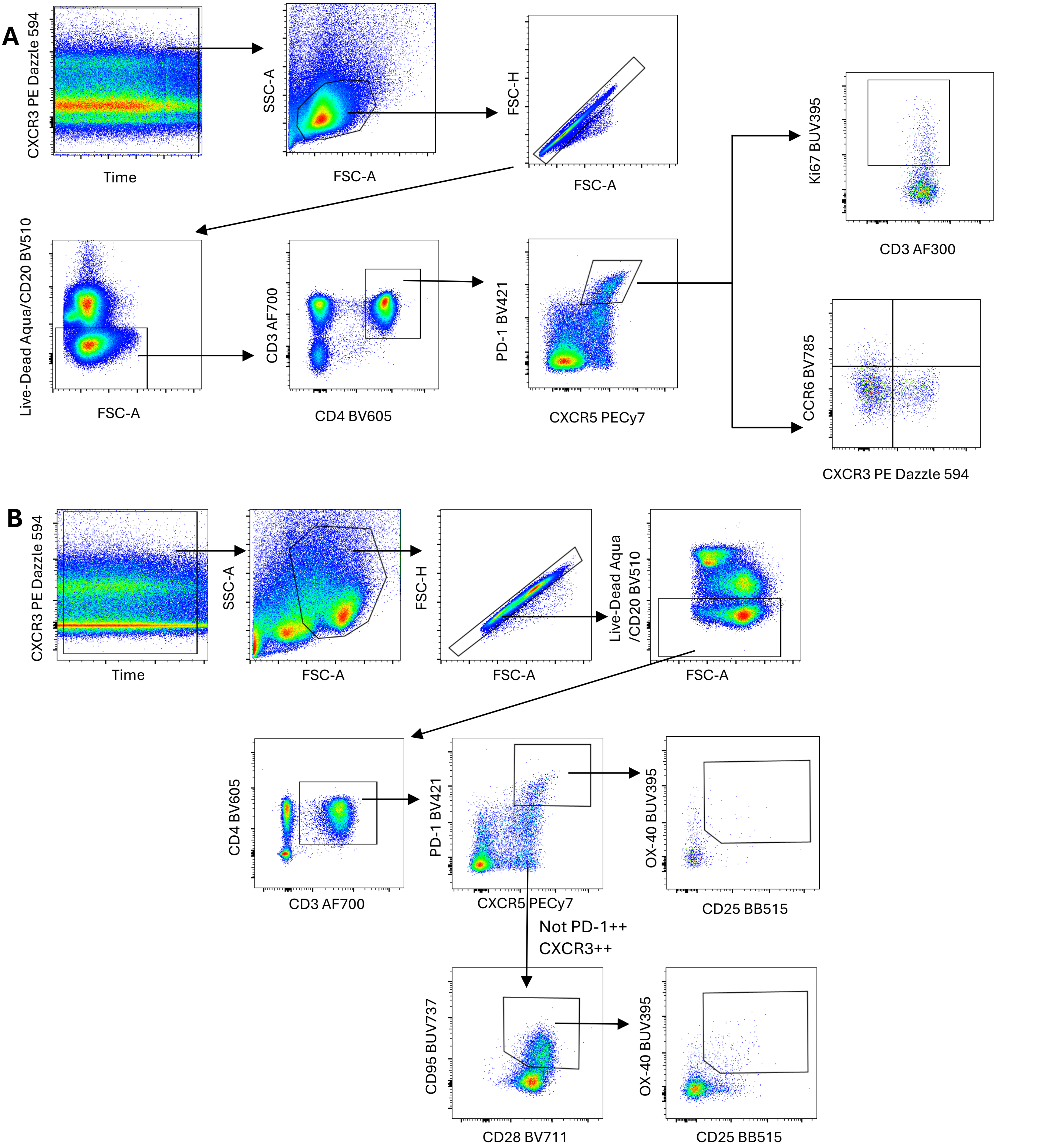

Figure S5. Identification of lymph node CD4 T cells in macaques. (A) TFH cells were first identified by Time, FSC-A vs SSC-A gating, followed by doublet exclusion (FSC-A vs FSC-H). Cells were then gated on CD20- live cells, followed by $CD3^{+}$ and $CD4^{+}$ expression. Bulk TFH cells were identified as $PD\text{-}1^{++}$ $CXCR5^{++}$, with proliferating TFH gated through $Ki67^{+}$. Th1 or Th17-like polarisation was further identified by CXCR3 and CCR6 expression. **(B)** Identification of antigen-specificity of TFH ($PD\text{-}1^{++}$ $CXCR5^{++}$) and TCM ($CD28^{+}$ $CD95^{+}$) subsets with similar upstream gating and by downstream gating of CD25 and OX-40 upregulation.
